# The factor structure of the Turkish version of the Revised Illness Perception Questionnaire (IPQ-R) in patients with diabetes and cardiovascular disease

**DOI:** 10.1186/1471-2458-12-852

**Published:** 2012-10-09

**Authors:** Patrick Brzoska, Yüce Yilmaz-Aslan, Eda Sultanoglu, Bülent Sultanoglu, Oliver Razum

**Affiliations:** 1 Department of Epidemiology & International Public Health, School of Public Health, University of Bielefeld, Bielefeld, Germany; 2 25 Aralık Government Hospital, Gaziantep, Turkey

**Keywords:** Revised illness perception questionnaire (IPQ-R), Turkish, Confirmatory factor analysis, Diabetes, Cardiovascular disease

## Abstract

**Background:**

The Revised Illness Perception Questionnaire (IPQ-R) has been used extensively in the study of illness perceptions across different populations. Only few confirmatory factor analytic (CFA) studies of the questionnaire are available. This study examines the construct and discriminant validity of the Turkish IPQ-R in patients with diabetes and cardiovascular disease focusing on the hypothesized seven dimensions of personal controllability, treatment controllability, timeline acute/chronic, timeline cyclical, coherence, consequences and emotional representations.

**Methods:**

302 patients (60.6% women) with a medically confirmed diagnosis of diabetes or cardiovascular disease and a mean age of 53.9 years were recruited from out-patient clinics in Turkey and surveyed by means of standardized interviews. Direct maximum likelihood confirmatory factor analysis was conducted.

**Results:**

Several areas of ill-fit were identified in the original measurement model of the IPQ-R. Four items (items 17, 19, 20, and 31) were deleted because of poor factor loadings. Also, two error covariances (between items 33 and 34 and between items 7 and 8) were added and item 6 respecified to obtain a good model fit. The modified 34-item model showed good reliability and discriminant validity.

**Conclusion:**

In accordance with studies on other language adaptations of the questionnaire, we identified certain items of the IPQ-R as potential sources of poor model fit. Their inclusion should be reconsidered in future applications of the questionnaire and researchers should examine whether our reduced set of items is stable across different populations. Our modified 34-item model showed a good reliability and discriminant validity and hence could be a valuable instrument in the assessment of illness perceptions in the Turkish health care setting, provided that the model is confirmed in subsequent research.

## Background

Chronic diseases such as diabetes and cardiovascular disease evolve gradually. They usually require long-term therapies and have consequences on the everyday life of affected individuals [1]. In the study of patients’ coping behavior and associated chronic disease outcomes, the self-regulatory model of illness (SRM) developed by Leventhal, Meyer & Nerenez has gained particular attention [2]. It suggests that individuals develop perceptions of their illness that have an impact on the choice and appraisal of coping strategies—and both illness perceptions and coping strategies are constantly adjusted to one another throughout the course of illness [2]. Leventhal et al. identified five core dimensions of cognitive illness perceptions: *identity* (perceptions about symptoms and illness labels), *consequences* (beliefs about possible illness effects), *timeline* (expectations about the duration of illness), *cause* (beliefs about the cause of the illness) and *cure/control* (beliefs about controllability and curability of the illness) [3]. These cognitive perceptions are accompanied by the development of emotional representations such as fear of illness severity.

The assumptions of the model are supported by empirical research. In a meta-analysis Hagger & Orbell illustrated that illness perceptions are associated with physical functioning, psychological distress, role functioning, social functioning, vitality, psychological well-being and health-related quality of life [4]. Also, there is some evidence that health outcomes and quality of care can be significantly improved when illness perceptions of patients are addressed in the health care process [5].

Among the quantitative instruments for the assessment of illness perceptions [6], the Illness Perception Questionnaire has gained most acceptance. It was originally developed by Weinman et al. [7] and was revised by Moss-Morris et al. [8] to improve its psychometric performance. Today the Revised Illness Perception Questionnaire (IPQ-R) is the most commonly used instrument for the assessment of illness perceptions. It is theoretically derived from the SRM with the dimensions of the SRM each being represented by a scale consisting of several items. In the course of the development of the IPQ-R, the control and timeline dimensions were further differentiated and each split into two scales. The *personal control* scale represents beliefs about personal abilities to control illness and the *treatment control* scale represents beliefs about the ability of the treatment or therapy to control or cure illness. Similarly, the *timeline acute/chronic* scale assesses beliefs about the duration of illness, while the *timeline cyclical* scale assesses beliefs about stability of illness symptoms over time. Moss-Morris et al. also introduced the *coherence* scale in order to measure the degree to which the individual considers his or her illness as comprehensive and clear [8].

Based on its layout and response format, the IPQ-R can be divided into three parts: *Part I* examining illness identity by means of 14 items with a double yes-no response format, *part II* assessing the seven dimensions of controllability (personal and treatment), timeline (acute/chronic and cyclical), coherence, emotional representations and consequences by means of 38 items with a 5-point Likert scale response format (strongly agree to strongly disagree), and *part III* applying the same response format and presenting 18 items on causal attributions.

Moss-Morris et al. [8] tested the validity and reliability of the IPQ-R in patients from eight different illness groups. It showed a good test-retest reliability and good divergent, known group and predictive validity. Exploratory analyses by means of principal component analysis supported the construct validity of the seven factor model of the IPQ-R part II.

Several translations of the instrument into other languages are available on the internet (http://www.uib.no/ipq). The Turkish version of the IPQ-R was evaluated exploratively by means of principal component analysis in patients with cancer and other physical diseases admitted to hospitals in Istanbul, Turkey [9]. It showed properties similar to the original version. Despite the extensive use of the IPQ-R in health research and practice, only few studies evaluated its factor structure by means of a hypothesis-testing approach. Confirmatory factor analysis (CFA), for example, is considered superior to data-driven procedures such as principal component and exploratory factor analysis. It allows to test for a factor structure that is hypothesized by theory and is powerful in exploring potential areas of ill-fit. These advantages are particularly important when researchers aim to examine the transferability of instruments across different populations and languages [10]. Aside from studies that aimed to confirm the validity of the IPQ-R applied to English speaking populations [11-16], to our knowledge only three CFA studies evaluated the factor structure of translated versions of the seven-dimensional core part of the IPQ-R (part II)—the Chinese [17], the Swedish [18] and an adapted Spanish version of the questionnaire [19]. All three CFA studies identified considerable sources of ill-fit in the measurement model proposed by Moss-Morris et al. [8] and several modifications had to be applied to obtain good model fit in the populations under study. These results illustrate that a thorough evaluation of the instrument’s construct validity is necessary before it can be applied in research on illness perceptions.

The aim of our study was to examine the factor structure of the Turkish IPQ-R and to test the measurement model proposed by Moss-Morris et al. [8] in Turkish patients with diabetes and cardiovascular disease by means of CFA. In line with previous research, we focused on the 38 items of the seven-dimensional IPQ-R part II.

## Methods

### Study design and data collection procedures

We recruited patients with a medically confirmed diagnosis of diabetes or cardiovascular disease (hypertensive disease, ischemic and pulmonary heart disease) from out-patient clinics in Gaziantep, Turkey during August 2010 and June 2011. Overall 314 patients were approached of which 302 (96.2%) participated in the study. Patients were surveyed by means of a quantitative questionnaire (see below) during their stay in the clinic using standardized interviews. They were informed about the purpose of the study and about the voluntary and anonymous nature of the data collection and analysis procedure. Prior to the survey, all participants gave consent to their participation. The study was cleared by the appropriate ethics committee at the University of Münster, Germany.

### Measures

The Turkish version of the Revised Illness Perception Questionnaire (part II) was administered as translated and published by Kocaman et al. [9] and Armay et al. [20]. The authors translated the questionnaire using a forward-backward translation by two independent researchers following guidelines of cross-cultural adaptation. It was pilot-tested in a clinical setting in Istanbul. In the present study, the original 5-point Likert scale response format was retained. Aside from the IPQ-R, the research instrument included questions on the duration of illness and on basic socio-demographic information such as age, sex and level of education.

### Sample size considerations

Given a number of ≥3 indicators per latent variable of the IPQ-R part II, a sample size of 300 can be considered sufficient for confirmatory factor analysis [21]. This was further supported by a Monte Carlo study with a simulated amount of 5% missing values conducted following published guidelines [22]. Population values for parameter estimates were obtained from prior research [9,12]. With a sample size of 300, parameter and standard error biases did not exceed the recommended threshold of 10%, coverage was between 93% and 97% and average values for goodness-of-fit indices indicated good model fit [22].

### Statistical analysis

Measures of central tendency, frequencies, chi-square-tests and independent t-tests were used for sample description.

Direct maximum likelihood (ML) confirmatory factor analysis (CFA) was used to examine the construct validity of the 38 items of the Turkish IPQ-R part II [10]. CFA was conducted using Mplus version 5.1 [23]. The hypothesized factor structure of the IPQ-R part II tested was the standard seven-factor solution identified by Moss-Morris et al. [8]. It consists of the timeline acute/chronic (items 1–5, 18), consequences (items 6–11), personal control (items 12–17), treatment control (items 19–23), illness coherence (item 24–28), timeline cyclical (items 29–32) and emotional representations (items 33–38) factor. The factors were allowed to covary.

The fit of the measurement model was assessed by different fit indices. The goodness-of-fit chi-square and the standardized root mean square residual (SRMR) were calculated as indices of absolute model fit. SRMR values ≤0.08 were considered to indicate acceptable model fit. The comparative fit index (CFI) and the Tucker-Lewis index (TLI) were used to evaluate the adequacy of the proposed CFA model in comparison to a restricted independence model. According to guidelines, CFI and TLI values >0.90 indicate a good fit of the CFA model. Also, the root mean square error of approximation (RMSEA) and its 90%-confidence interval were calculated with RMSEA values between 0.05 and 0.08 considered indicating a reasonable and values <0.05 considered indicating a good model fit [10,24].

The reliability was assessed by composite reliability estimates (ρ) with values ≥0.60 indicating satisfactory reliability in the latent factors [25]. Discriminant validity of the seven factors was evaluated by the size of their intercorrelations with correlation coefficients <0.85 indicating acceptable discriminant validity [24].

Items with completely standardized factor loadings (λ) <0.40 and standardized residuals >2.58 were considered for deletion. Furthermore, modification indices (MI) and completely standardized expected parameter changes (EPC) were calculated in order to identify potential for model improvement. Only modifications reasonable on theoretical grounds were made and implemented one-by-one as suggested by published guidelines [10].

A maximum of 3.8% missing values was observed for the IPQ-R items in the sample. To prevent selection bias, direct ML estimation was used for CFA as recommended by Brown [10].

## Results

### Sample description

302 patients with diabetes and/or cardiovascular disease participated in the study. Of these, 183 (60.6%) were female. On average, patients were 53.9 years old, with a mean duration of illness treatment of 8.8 years. Men had a significantly higher educational status than women, with 8.5% of men as compared to 37.4% of women having no formal school education (p < 0.001). 62.1% of all patients reported to have diabetes, 20.6% stated to have cardiovascular disease and 17.3% reported both conditions. These proportions slightly differed between men and women (p < 0.05). Basic characteristics of the study sample stratified by sex are displayed in Table 1.

**Table 1:** Basic characteristics of the sample stratified by sex

	**Sex**		**Total (n = 302)**
	**Male (n = 119)**	**Female (n = 183)**	
**Age** in years (mean; sd) (p > .05)	55.4; 11.8	52.9; 10.9	53.9; 11.3
**Education** (%)(p < .001)			
no school education	8.5	37.4	26.1
primary school education	47.9	41.2	43.8
secondary school education	11.1	5.5	7.7
high school or university education	32.5	15.9	22.4
**Diseases** (%) (p < .05)			
Diabetes	58.5	64.5	62.1
Cardiovascular disease	27.1	16.4	20.6
Diabetes and cardiovascular disease	14.4	19.1	17.3
**Duration of disease treatment** in years (mean; sd) (p > .05)	8.9; 6.6	8.7; 6.7	8.8; 6.6

*sd*: standard deviation.

### Confirmatory factor analysis

The original factor structure proposed by Moss-Morris et al. [8] did not fit the data well as indicated by several goodness-of-fit measures. Although RMSEA indicated a reasonable fit (RMSEA = 0.075 [90%-CI = 0.070-0.079]), the other fit indices were below the thresholds for acceptable fit (*χ*^2^ = 2396.73 [df = 644,p < 0.001]; SRMR = 0.109; TLI = 0.765; CFI = 0.785; AIC = 18177.43). Four items showed low and non-significant factor loadings. The first was purported to measure personal control (item 17 “My actions will have no effect on the outcome of my illness”, λ=0.11), the second and third were part of the treatment control factor (item 19 “There is little I can do to control my illness”, λ=0.10; item 20 “Treatment will be effective in treating my illness”, λ=0.19) and the fourth was specified to load on the timeline cyclical factor (item 31 “My illness is very unpredictable”, λ=0.21). All other factor loadings were significant and ranged from 0.40 to 0.90. All standardized residuals were <2.58.

For a modified model (model 2a), we dropped the above mentioned items with poor loadings and conducted CFA on the remaining 34 items. Goodness-of-fit indices suggested an improvement of the model fit as compared to the initial model (*χ*^2^ = 1479.46 [df = 506,p < 0.001]; RMSEA = 0.059 [90%-CI = 0.054-0.064]; SRMR = 0.083; TLI = 0.864; CFI = 0.877; AIC = 15404.43). However, fit indices were still not within levels that indicate acceptable fit. Also, modification indices (MI) and completely standardized expected parameter changes (EPC) suggested that relevant modifications could be made in the model. As these modifications were theoretically sound they were carried out in a second modified model (model 2b) and implemented one-by-one. The two largest modification indices suggested to add error covariances between items 33 (“I get depressed when I think about my illness”) and 34 (“When I think about my illness I get upset”) belonging to the emotional representations factor (MI = 109.571; EPC = 0.158) as well as between items 7 (“My illness has major consequences on my life”) and 8 (“My illness does not have much effect on my life”) belonging to the consequences factor (MI = 80.522; EPC = 0.845). The respecified model resulted in a considerably better fit (*χ*^2^ = 1261.09 [df = 504,p < 0.001]; RMSEA = 0.050 [90%-CI = 0.045-0.056]; SRMR = 0.079; TLI = 0.901; CFI = 0.911; AIC = 15189.58). Finally, MI and EPC suggested to respecify item 6 (“My illness is a serious condition”) to load on the timeline acute/chronic factor (MI = 72.503; EPC = 0.690). The revised final model (model 2c) with 34 indicators and two error covariances resulted in good fit suggesting superiority to the other three models (*χ*^2^ = 1150.39 [df = 504,p < 0.001]; RMSEA = 0.045 [90%-CI = 0.039-0.050]; SRMR = 0.067; TLI = 0.921; CFI = 0.929; AIC = 15079.40). A detailed overview of the goodness-of-fit indices for the four models is presented in Table 2.

**Table 2:** Goodness-of-fit indices for the original and revised models of the Turkish IPQ-R part II (n = 302)

**Model**	***χ***^**2**^**(df)**	**Δχ**^**2**^**(Δdf)**	**RMSEA (90%-CI)**	**SRMR**	**TLI**	**CFI**	**AIC**
Model 1 (M1): Original (38 items)	2396.73		0.075	0.109	0.765	0.785	18177.43
	(644)*		(0.070-0.079)				
Model 2a (M2a): Modified (34 items)	1479.46	M1-M2a:	0.059	0.083	0.864	0.877	15404.43
	(506)*	917.27 (138)*	(0.054-0.064)				
Model 2b (M2b): Modified (34 items, 2 error covariances)	1261.09	M2a-M2b:	0.050	0.079	0.901	0.911	15189.58
	(504)*	218.37 (2)*	(0.045-0.056)				
Model 2c (M2c): Modified (34 items, 2 error covariances, item 6 respecified)	1150.39	M2b-M2c:	0.045	0.067	0.921	0.929	15079.40
	(504)*	110.70 (0)	(0.039-0.050)				

Note*. χ*^*2*^*(df)*: chi-square test of model fit and degrees of freedom; *RMSEA (90%-CI)*: root mean square error of approximation and 90%-confidence intervals; *SRMR*: standardized root mean square residual; *CFI*: comparative fit index; *TLI*: Tucker-Lewis index: *AIC*: Akaike Information Criterion; *p < 0.001.

Table 3 shows completely standardized factor loadings, standard errors and composite reliabilities for the revised final model (model 2c). All factors were statistically significant and composite reliability estimates exceeded the recommended threshold of 0.60, indicating satisfactory reliability in the latent factors.

**Table 3:** Results of confirmatory factor analysis of the modified final measurement model (Model 2c: 34 items, 2 error covariances, item 6 respecified) of the Turkish IPQ-R part II

**Factors and items**	**λ**	**SE**	**ρ**
*Timeline acute/chronic*			0.92
1: My illness will last a short time	0.92**	0.03	
2: My illness is likely to be permanent rather than temporary	0.95**	0.02	
3: My illness will last a long time	0.96**	0.02	
4: This illness will pass quickly	0.89**	0.04	
5: I expect to have this illness for the rest of my life	0.96**	0.02	
18: My illness will improve in time	0.76**	0.05	
6: My illness is a serious condition	0.63**	0.06	
*Consequences*			0.81
7: My illness has major consequences on my life	0.58**	0.06	
8: My illness does not have much effect on my life	0.49**	0.06	
9: My illness strongly affects the way other see me	0.61**	0.05	
10: My illness has serious financial consequences	0.71**	0.05	
11: My illness causes difficulties for those who are close to me	0.79**	0.04	
*Personal Control*			0.82
12: There is a lot I can do to control my illness	0.57**	0.07	
13: What I do can determine whether my illness gets better or worse	0.84**	0.05	
14: The course of my illness depends on me	0.77**	0.07	
15: Nothing I do will affect my illness	0.55**	0.09	
16: I have the power to influence my illness	0.82**	0.06	
*Treatment Control*			0.63
21: The negative effects of my illness can be prevented by my treatment	0.63**	0.18	
22: Treatment can control my illness	0.56*	0.22	
23: There is nothing that can help my illness	0.48**	0.14	
*Illness Coherence*			0.91
24: The symptoms of my illness are puzzling to me	0.61**	0.06	
25: My illness has no meaning to me	0.87**	0.03	
26: I don’t understand my illness	0.90**	0.03	
27: My illness doesn’t make any sense to me	0.90**	0.03	
28: I have a clear picture or understanding of my illness	0.85**	0.04	
*Timeline cyclical*			0.61
29: The symptoms of my illness change from day to day	0.49**	0.06	
30: My symptoms come and go in cycles	0.63**	0.09	
32: I go through cycles in which my illness gets better and worse	0.87**	0.09	
*Emotional representations*			0.94
33: I get depressed when I think about my illness	0.88**	0.03	
34: When I think about my illness I get upset	0.90**	0.02	
35: My illness makes me feel angry	0.58**	0.05	
36: My illness does not worry me	0.86**	0.03	
37: My illness makes me feel anxious	0.95**	0.01	
38: My illness makes me feel afraid	0.89**	0.02	
**Error covariance between items 33 and 34**	0.69**	0.09	
**Error covariance between items 7 and 8**	0.82**	0.04	

Completely standardized factor loadings, standard errors, composite reliabilities and error covariances; n = 302; Note. λ: completely standardized factor loading; *SE*: standard error; ρ: composite reliability; *p < 0.01, **p < 0.001.

Correlations between the seven latent factors of the IPQ-R part II are given in Table 4. No intercorrelation exceeded the threshold of 0.85, suggesting acceptable discriminant validity. The largest correlations were observed between the treatment and personal control factor (r = 0.54), between the consequences and emotional representations factor (r = 0.46) and between the timeline acute/chronic and consequences factor (r = 0.36).

**Table 4:** I**ntercorrelations between the seven latent factors of the Turkish IPQ-R part II (n = 302)**

**Factors**	**1**	**2**	**3**	**4**	**5**	**6**	**7**
1. Timeline acute/chronic							
2. Consequences	0.36**						
3. Personal control	0.29*	0.03					
4. Treatment control	0.13	0.00	0.54*				
5. Illness coherence	0.13	0.03	0.29*	0.10			
6. Timeline cyclical	0.31*	0.24**	0.02	0.21	−0.08		
7. Emotional representations	0.22**	0.46**	−0.07	−0.10	−0.25**	0.25**	

Note. *p < .01; **p < .001.

## Discussion

The aim of our study was to formally assess the factor structure of the Turkish IPQ-R and to rigorously test the measurement model proposed by Moss-Morris et al. (2002) [8] in Turkish patients with diabetes and cardiovascular disease residing in Turkey. Aside from this study, only three other studies have examined the dimensional structure of non-English language versions of the IPQ-R by means of a hypothesis-testing framework. The original structure of the second part of the IPQ-R could not be confirmed for the population in our study. While the emotional representations and illness coherence factor showed satisfactory construct validity, several modifications had to be applied to the other factors to achieve a good model fit. A poor fit of the original measurement model was also observed in other CFA studies. This comprises an evaluation of the Chinese IPQ-R applied to hypertensive patients in Taiwan [17], an evaluation of the Swedish IPQ-R applied to patients recovering from myocardial infarction [18] as well as several CFA studies on the English version of the instrument applied to patients with different chronic diseases [11-13,15,16], including a recent study on populations of African origin with type 2 diabetes [14]. In these studies, several areas of ill-fit were identified and substantial changes to the measurement model such as the deletion of items and the respecification of indicators had to be applied to achieve acceptable model fit.

In our study, items purported to load on the personal control, treatment control and timeline cyclical factor had to be deleted from further analysis due to low factor loadings. Two of these four items (17 and 19) are negatively worded and, for the same reason, were also dropped from the analysis in the studies by Chen et al. [17] on the Chinese and Cabassa et al. [19] on a shortened 27-item Spanish version of the IPQ-R. Though retained in the model, item 17 also showed a low factor loading (standardized λ=0.25) in the Swedish validation study of the IPQ-R [18]. Item 19 was also deleted by Abubakari et al. [14] in their study of the IPQ-R in type 2 diabetes patients of African origin because of a low loading on its respective factor. The problems encountered with these two items may be due to a method bias. It is known from scale development research that negatively worded items may introduce method effects because they can be misunderstood by respondents [26].

Similar to our study, item 20 was also considered problematic by Abubakari et al. [14]. Based on consultations with subjects from their study population, the authors argue that this may be due to a mismatch between own beliefs of the patients (that are also shaped by supernatural causal attributions) and information they receive from others (e.g. doctors). This may be also true for Turkish patients who have been reported to have strong beliefs in fate and divine influence [27,28].

The four items (17, 19, 20, 31) deleted in our study should be thoroughly scrutinized in further applications of the Turkish IPQ-R and their omission should be considered. Researchers should also thoroughly evaluate the reliability of the timeline cyclical and treatment control factor. Though still above the threshold that indicates acceptable reliability, the composite reliability of both factors was lower than for the other five factors of the model.

Modifications indices strongly suggested to add two error covariances between items 7 and 8 and between items 33 and 34, indicating that these item pairs shared measurement error. Similar to the reversed coding of items 17 and 19, the error covariance between items 7 and 8 may result from the negative wording used in item 8. The error covariance between items 33 and 34 most likely results from a very similar phrasing (see Table 3) which in the Turkish version only differs by a weakly discriminating adjective (“Hastalığımı düşündüğüm zaman *çökkün* oluyorum” vs. “Hastalığımı düşündüğüm zaman *üzgün* oluyorum”). In addition, unlike *üzgün* the word *çökkün* is rather rarely used in the Turkish language which bears potential for additional bias.

We recommend an alternative wording for item 33 such as “Hastalığımı düşündüğüm zaman bunalıyorum” or “Hastalığımı düşündüğüm zaman karamsar oluyorum” (likewise literally meaning “When I think about my illness I get depressed“) that may be better able to discriminate and achieve equivalence with the original questionnaire.

In our study, item 6—originally purported to load on the consequence factor—was related to the timeline acute/chronic factor. Presumably this is because of the item’s wording used in the Turkish version (“Ciddi bir hastalığım var”). Although the adjective *ciddi* means “serious”, it is often associated with chronicity in the context of illness. The adjective *ağır* (severe, serious) may be better able to attain semantic equivalence with the original version and should be tested in future evaluations of the Turkish version.

The intercorrelations observed between the factors of the revised final model support the discriminant validity of the latent dimensions and are mostly in accordance with those reported in other studies on the IPQ-R [8,12,14,17,18]. The largest correlations indicated that patients who perceive their treatment as effective also hold stronger beliefs about personal abilities to control their illness. Similarly, individuals viewing their illness as a condition with serious consequences also show a more intense emotional response to their illness. Both findings are in line with assumptions of the self-regulatory model [3].

The present study has limitations. First, our results cannot be generalized to patients with diseases other than diabetes and cardiovascular disease. Second, the post hoc modifications of the model that we made guided by modification indices and theoretical considerations have to be considered an exploratory type of analysis [10]. Thus, the changes we suggested are a starting point for further analysis that should aim to cross-validate and confirm the revised measurement model for other populations. Third, we are aware that a sample size of 300 is smaller than suggested by classical rules of thumb that are usually guided by the ratio of subjects to model parameters [25]. However, these recommendations have to be reconsidered in light of newer simulation studies [21,29]. They show that characteristics such as the number of indicators in the model, the number of indicators per latent variable and the average size of factor loadings are of greater importance to attain precise parameter estimates. Given the high factor loadings in the present study (on average factor loadings in the measurement model were ≥0.70) and a ratio of indicators per latent variable of at least 3:1, the estimation in the present study can be considered robust. In order to further support the precision of our results we conducted an additional Monte Carlo simulation using the model estimates as population values following suggestions by Brown [10]. The results showed that with the given sample size and missing values proportion, the percentage of parameter and standard error bias, the degree of coverage and average values for goodness-of-fit indices were within acceptable ranges [10,22].

## Conclusion

Our study adds to current research on illness perceptions by providing a thorough examination of the factor structure of the Turkish Revised Illness Perception Questionnaire using confirmatory factor analysis. We identified several misspecifications that contributed to a poor model fit in the model proposed by the developers of the original IPQ-R and—similar to studies on other language adaptations of the questionnaire—some modifications had to be applied before a good model fit could be achieved. We can confirm the observations made in other studies on the IPQ-R that certain items are potential sources of ill-fit. The inclusion of these items should be reconsidered in future applications of the instrument and researchers should examine whether our reduced set of items is stable across different populations. The modifications we applied to the original measurement model could serve as a starting point for further analysis. In this respect, it must also be noted that despite the fact that the Turkish translators of the IPQ-R used a rigorous translation and adaptation procedure including pilot testing [9,20], the translation remains rather literal and applies standard rather than spoken language. Since both aspects may reduce the questionnaire’s semantic equivalence to the original version, we recommend that the further evaluation of the Turkish IPQ-R also comprises a qualitative component. For instance, this may include a think-aloud survey on the understanding, interpretation and usability of the questionnaire [30]. Similar surveys were for example conducted on the Brief IPQ [31].

Our modified 34-item model showed a good reliability and discriminant validity and hence could be a valuable instrument in the assessment of illness perceptions in the Turkish health care setting, provided that the model is confirmed in subsequent research. Given the role of illness perceptions in clinical practice, we therefore strongly encourage further evaluation of the questionnaire in health research and practice. The IPQ-R may be also useful in the study of illness perceptions among Turkish migrant populations and could help to improve health care for this population group in different host countries. This could address findings from health services research showing that Turkish migrant patients—similar to other ethnic minority groups—experience barriers to health care access and effectiveness that may result from culture-specific illness perceptions [32,33]. Prior to an application, however, a separate validation of the IPQ-R in these populations is necessary [34].

## Competing interests

The authors declare that they have no competing interests.

## Authors’ contributions

PB developed the concept and design of the study, performed the statistical analysis, interpreted the findings and drafted the manuscript. YYA contributed to the development of the study design and research instrument, trained the interviewer (ES) and helped to interpret the findings and to write the manuscript. ES conducted the standardized interviews and together with BS coordinated the field work. OR helped with the study design and data interpretation and critically revised the manuscript. All authors read and approved the final manuscript.

## Pre-publication history

The pre-publication history for this paper can be accessed here:

http://www.biomedcentral.com/1471-2458/12/852/prepub
